# Interglobular dentine attributed to vitamin D deficiency visible in cremated human teeth

**DOI:** 10.1038/s41598-021-00380-w

**Published:** 2021-10-25

**Authors:** Barbara Veselka, Christophe Snoeck

**Affiliations:** 1 Maritime Cultures Research Institute, Department of Art Sciences and Archaeology, Vrije Universiteit Brussel, Brussels, Belgium; 2 Research Unit: Analytical, Environmental & Geo-Chemistry, Department of Chemistry, Vrije Universiteit Brussel, Brussels, Belgium; 3 G-Time Laboratory, Université Libre de Bruxelles, Brussels, Belgium

**Keywords:** Microscopy, Anatomy, Diseases

## Abstract

Vitamin D deficiency has hugely impacted the health of past societies. Its identification in skeletal remains provides insights into the daily activities, cultural habits, and the disease load of past populations. However, up till now, this approach remained impossible in cremated bones, because temperatures reached during cremations destroyed all macroscopic evidence of vitamin D deficiency. This precluded the analyses of a large fraction of the archaeological record, as cremation was an important burial ritual from the Late Neolithic until the Early Medieval period in Europe. Here, the identification of interglobular dentine (IGD), a dental mineralisation defect attributed to vitamin D deficiency, in experimentally burnt teeth, demonstrates this deficiency to be observable in human teeth burned to temperatures as high as 900 °C. In most cases, it becomes even possible to assess the ages-of-occurrence as well as the severity of the IGD and possibly vitamin D deficiency intensity. This study represents a major step forward in the fields of biological anthropology, archaeology, and palaeopathology by opening up a variety of new possibilities for the study of health and activities related to sunlight exposure of numerous past populations that practiced cremation as their funerary ritual.

## Introduction

The investigation of palaeopathological conditions fundamentally increases our understanding of a wide range of sociocultural variables, such as past living conditions^1,2^, child rearing practices^3,4^ dietary transitions^5^, gendered division of labour^6,7^, social inequality^1,4^, and other factors^8–10^. Yet, these palaeopathological conditions remain almost exclusively identified on inhumed individuals. Although the osteological study of cremated human remains has tremendously increased over the years, opening new possibilities, most of these studies have focused on improving sexing^11–13^ and ageing methods^14–16^ since biological sex and age are vital data needed for the reconstruction of both an individual’s identity as well as the social organization of past populations. In parallel, cremated human remains are also often included in more recent palaeomobility studies using strontium isotope ratios^17–22^. However, palaeopathological studies of cremated human bones remains scarce^21,23^.

Indeed, due to the high degree of fragmentation and the macro- and microscopic changes to the bones caused by the burning process, part of the biological information that can usually be obtained from the unburnt human skeleton is lost, in particular data on palaeopathological conditions^24,25^. In addition, preferably the whole skeleton needs to be investigated to enable an accurate interpretation of the lesions observed^26,27^, but individuals buried in cremation deposits are often incomplete, further hindering the evaluation of pathological anomalies. Pathological lesions that, so far, have been observed in cremated remains are non-specific stress indicators, such as diffuse porosity of the cranial vault and orbital roofs^21,28^ or degenerative joint diseases^29^. In some cases, pathological fractures have also been identified^23^. However, a lacuna in our knowledge of the occurrence of the majority of pathological conditions, such as vitamin D deficiency, in populations that practiced cremation crucially limits our understanding of sociocultural habits, daily activities, and health. Since cremation was a widespread funerary practice from the beginning of the Metal Ages until the Early Medieval period in large parts of Europe^30–33^, cremation deposits constitute an important part of past human remains that so far precluded the evaluation of pathological conditions that document the disease load and development, sociocultural practices (e.g. child rearing practices; diet), and socioeconomic characteristics (e.g. subsistence strategies; level of urbanisation).

A complex interplay of biophysical variables cause vitamin D deficiency, including geographical latitude (e.g. solar zenith) and the naturally available amounts of vitamin D in foods, as well as sociocultural variables, such as child rearing practices and daily activities related to sunlight exposure^34^. Especially differences in sociocultural variables likely influence the access to high quality foods, the amount of time spent outdoors, and the type of clothing worn^34,35^. As such, the occurrence of vitamin D deficiency provides vital information on sociocultural variables needed for the reconstruction of the social organisation of past populations^3,4,6,36^. Until recently, most vitamin D deficiency cases were diagnosed based on a number of established macroscopic features that provide information on the presence or absence and, in some cases, the stage (active vs. healed) of the disease^37^. Recent studies by D’Ortenzio et al.^38^ and Veselka et al.^3^ investigated the occurrence of interglobular dentine (IGD), a mineralisation defect of the dentine attributed to vitamin D deficiency, by microscopically evaluating the presence of this defect in unburnt teeth and using its presence as a proxy for determining vitamin D deficiency prevalence. In addition, the number of deficient episodes, the severity of the defect, and the ages of disease occurrence was investigated. This information cannot be obtained by macroscopic assessment and the microscopic evaluation of IGD via thin sections is currently the only way to provide evidence for vitamin D deficiency in fragmented or incomplete human remains, where macroscopic assessment is difficult or even impossible. Consequently, this approach represents the only way to study vitamin D deficiency in cremated human remains. There has, however, been no attempt so far to assess this due to the reported changes to bone and teeth caused by high temperatures^39–41^. Yet, the detection of vitamin D deficiency in cremated remains aids in reconstructing sociocultural variables influencing daily life of past individuals, especially from populations for which only little to no contextual information is available (e.g. Bronze and Iron Age).

This study assesses the visibility of IGD in burnt dentine. This is achieved by experimentally burning teeth at various temperatures and durations. By demonstrating that IGD is still observable in cremated teeth, our study not only demonstrates vitamin D deficiency detection to be possible in cremated human remains, which until now was deemed unattainable, but also creates new opportunities in several scientific fields to further improve our understanding of daily life and other aspects of human behaviour (e.g. pyre management; disease load) in past and modern populations.

## Results

**Figure 1:**
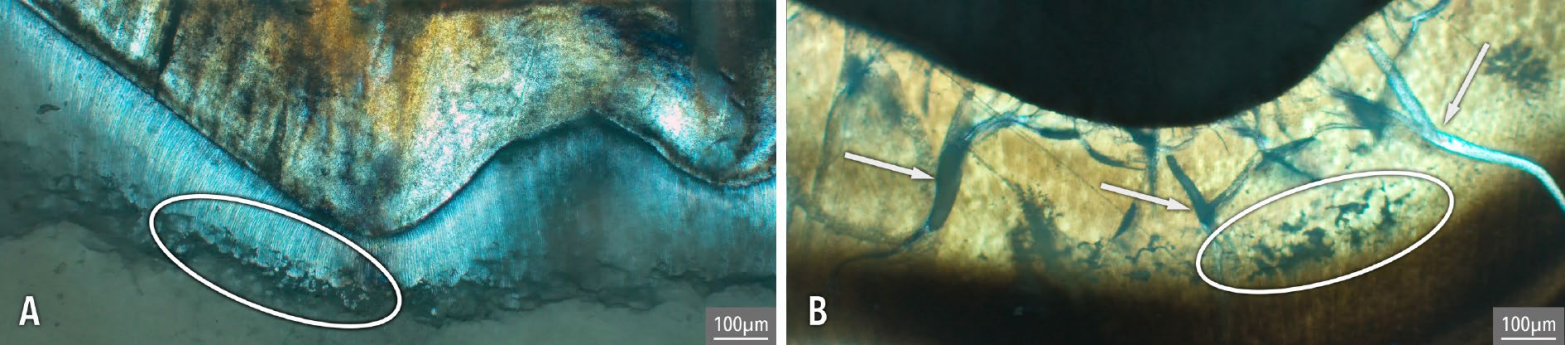
IGD (white ovals) in unburnt RM1 (**A**) and burnt LM1 (**B**) from B121 at approximately the same location. Note the heat-induced cracks in LM1 (see arrows).

A total of 17 paired teeth from the archaeological collections of Koekelberg (Belgium) and Zwolle (the Netherlands) were analysed for IGD by two different researchers: one with osteoarchaeological experience (BV) and one without (CS—Table 1). For each pair of teeth, one was analysed unburned, while the other was burned in a muffle furnace. No IGD was observed in the unburnt nor burnt deciduous molars from B025, implying that no feature mimicking the presence of IGD developed as a result of the heating process. Molars B090, B015, B097, B045, B030, and B047 (6/17; 35.3%) fell apart during burning, inhibiting IGD evaluation. An additional four teeth were considered unobservable by observer CS, due to the level of diagenesis or high degree of fragmentation. For all other teeth (unburnt = 17; burnt = 11), it was possible to make thin sections and assess IGD prior to and after burning (Fig. 1).

**Table 1:**
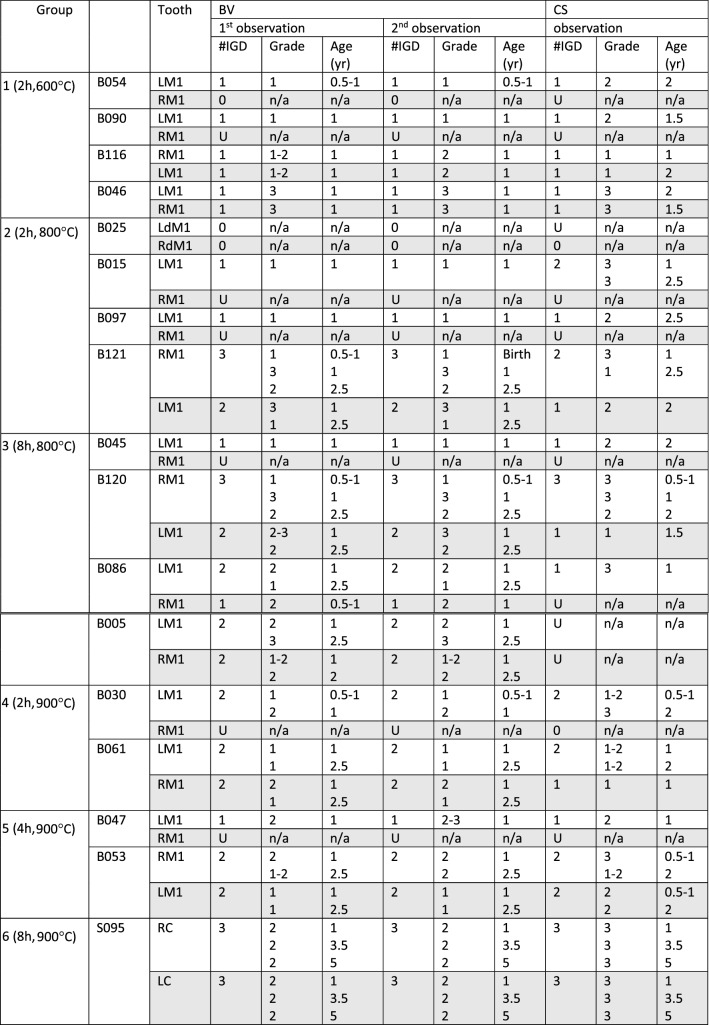
IGD occurrence per tooth noted for the 1st and 2nd observation of BV and the observation of CS per group. The grey boxes represent the burnt teeth.

LM1 = left permanent first molar, RM1 = right permanent first molar, LdM1 = left deciduous first molar, RdM1 = right deciduous first molar, U = unobservable, n/a = not applicable, RC = right mandibular canine, LC = left mandibular canine.

All of the teeth (burnt and unburnt) yielded the same number of IGD bands in both observations from BV. In the burnt molars of B054, B121, B120, B086, one episode of IGD was not observed in the second observation of BV (36.4%; 4/11), which was graded 1 in the unburnt molars, suggesting that IGD graded 1 may not always be visible in burnt molars. A difference in IGD grading between the unburnt and burnt molar of B005, B053, and B121 was observed in BV’s observations (27.3%; 3/11), whereby IGD was scored as being less severe, while in B061, IGD was scored as more severe (9.1%; 1/11). Age estimates for the observable episodes were the same in the unburnt and burnt molars.

Considering only the observable teeth, in most of the unburnt teeth (73.3%; 11/15), CS scores the same number of IGD episodes as BV, whereby in B086 and B121, the band graded 1 is absent in the observation of CS. Instead, diagenetic alterations mimicking IGD is scored as IGD Grade 2 or 3 in B054 and B015 respectively, explaining the difference in IGD severity between BV and CS. Differences in severity scores between CS and BV exist in the unburnt molars, whereby about half of the IGD bands receives different scores. Age estimations between CS and BV match for 73.3% (11/15). The number of IGD bands and IGD severity in more than half of the burnt teeth (62.5%; 5/8) is scored the same in both BV’s and CS’ observations. Age estimation is scored 87.5% (7/8) similarly. Based on the results of BV’s observations, the number of IGD bands and severity is similarly scored in the unburnt and burnt teeth.

**Figure 2:**
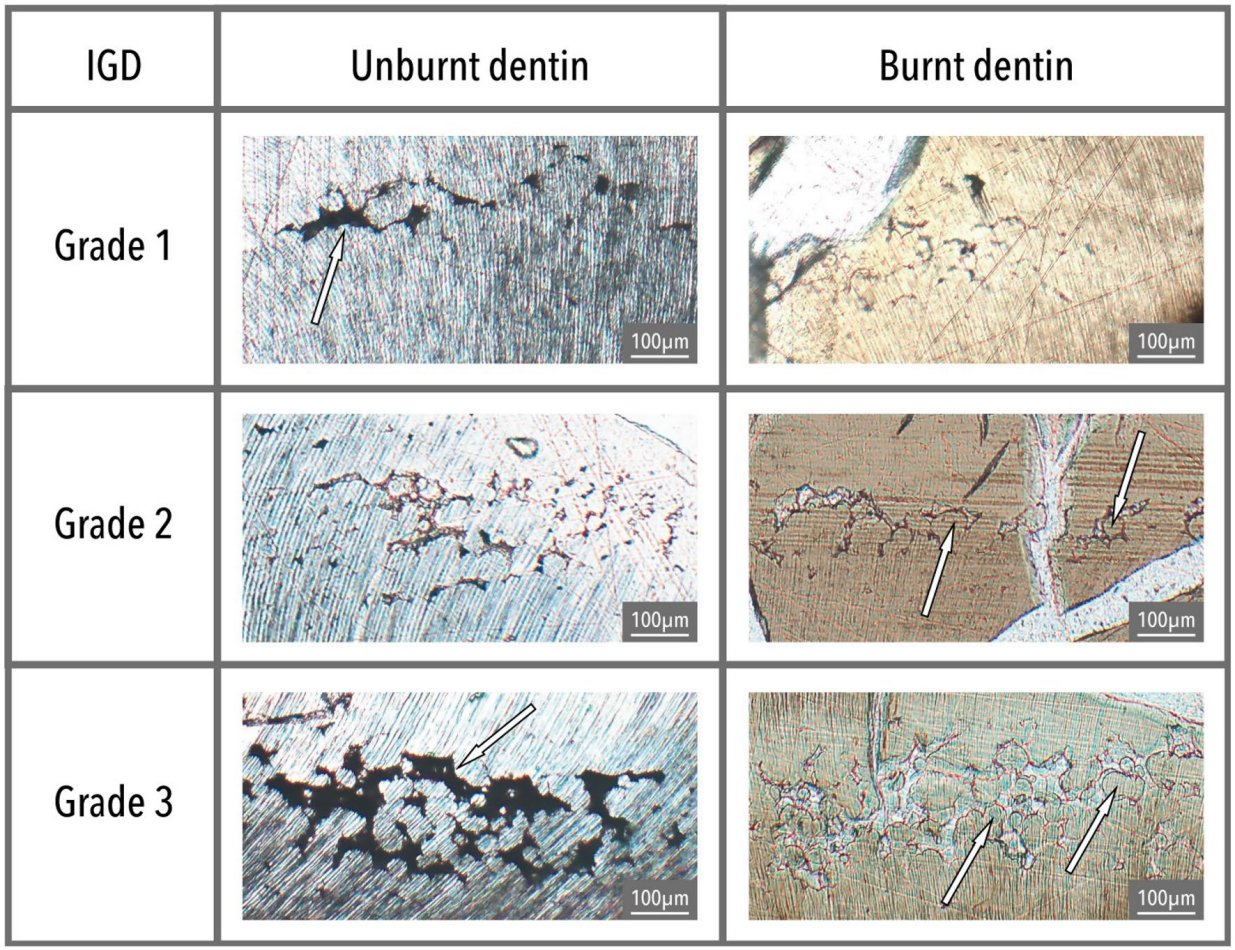
IGD appearance in unburnt (dark semi-circular spaces, see arrows) and burnt dentine (separately visible globules, calcospherites, see arrows). Burnt dentine Grade 1 is depicted in dentine burned at 800 °C (2 h), visible as dark semi-circular spaces, Grade 2 in dentine burned at 900 °C (2 h), and Grade 3 in dentine burned at 600 °C (2 h).

**Figure 3:**
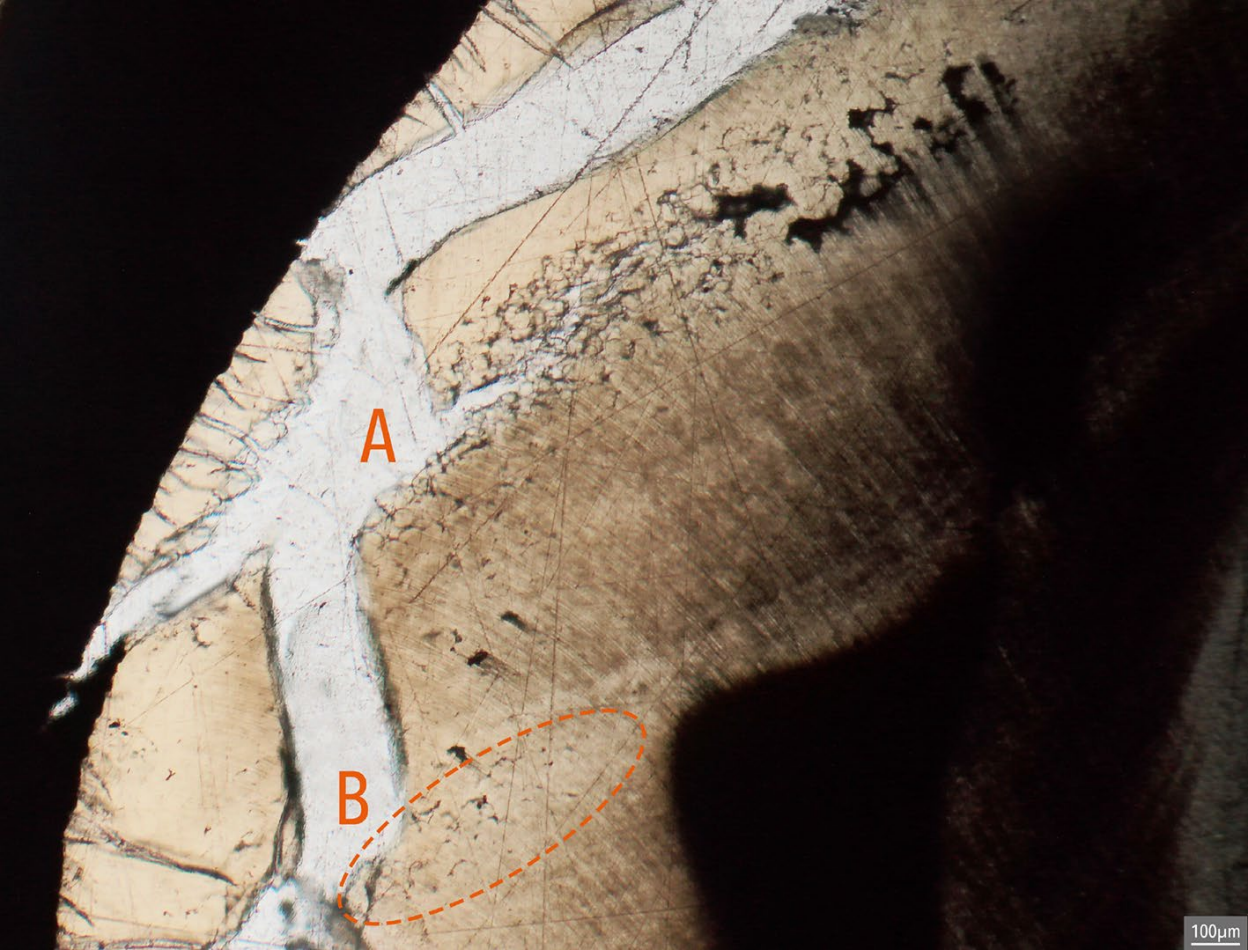
Two bands of IGD in the permanent left 1st mandibular molar from B121 after burning at 800 °C for 2 h. Band A has Grade 3 and shows both partially fused calcospherites (close to the A) as well as the dark semi-circular spaces towards the end of the band. Band B (see orange oval) has Grade 1. Note the heat-induced damage (white cracks) to the dentine (light brown).

**Figure 4:**
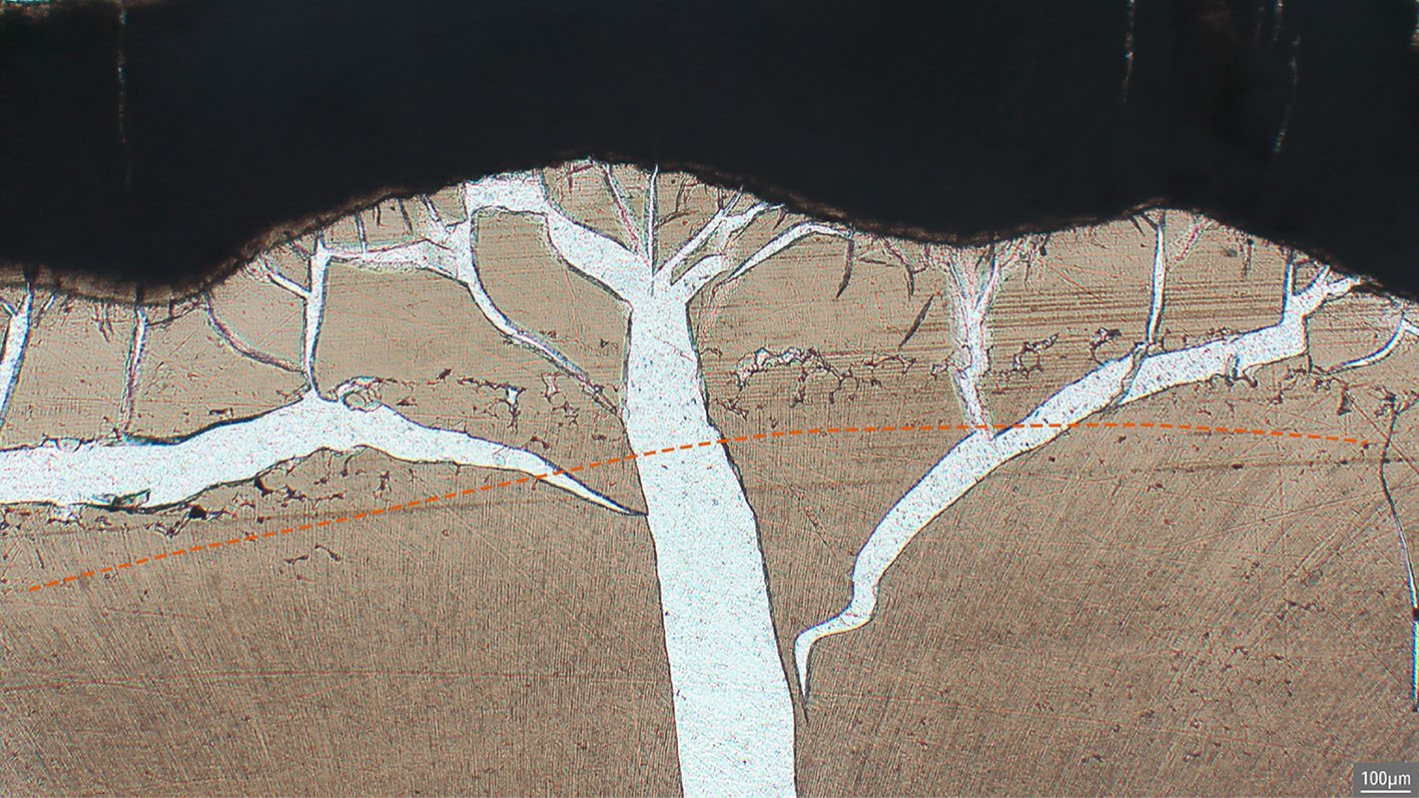
IGD in B61 (Grade 2) burned at 900 °C for 2 h, visible as a band of not fully fused calcospherites just above the dashed orange line. Note the heat-induced damage (white cracks) to the dentine (light brown).

Regardless of severity, IGD mostly appears as dark semi-circular spaces in unburnt dentine. In burnt dentine, IGD may have the same appearance as in unburnt dentine or present itself as partially or unfused calcospherites, which are spherically shaped calcium salts (Figs. 2 and 3)^38^. The presence of IGD seems to weaken the dentine, thereby possibly increasing the risk of heat-induced cracking at the location of the IGD, resulting in the partially fused calcospherites being visible at the border of such a crack (Fig. 3). Similarly, while some of the dental microstructures are clearly lost in the teeth burned at 900 °C, IGD is still visible as a band of partially fused calcospherites (Fig. 4).

## Discussion

For the first time, this study clearly shows that IGD is clearly visible in burnt human teeth at temperatures as high as 900 °C regardless of duration. The appearance of IGD in burnt teeth remains similar to that in unburnt teeth (see Fig. 2), whereby often a combination of dark semi-circular spaces (i.e. how IGD usually appears in unburnt dentine) and separately visible calcospherites are observable (Fig. 3). In dentine burned at 900 °C, regardless of duration, the morphology of IGD seems to solely consist of partially fused calcospherites (Fig. 4). Therefore, partially fused calcospherites likely constitute the characteristic feature to look for in burnt dentine when evaluating cremated human remains for vitamin D deficiency. Although the amount of heat-induced damage and the completeness of the burnt tooth may influence IGD assessment, our results suggest that the lower ends of Grade 1 may not always be observable in burnt dentine, which is also the case in unburnt teeth evaluated via µCT scan^3,42^. This potentially results in an underrepresentation of the number of vitamin D deficient individuals. Yet, all the IGD bands with Grades 2 and 3 were observable in burnt teeth regardless of temperature or duration. Our result show that the severity and the ages-of-occurrence are scored similarly in both the burnt and unburnt teeth (see Table 1; observations BV), implying that IGD is visible in burnt dentine and can easily be detected, although training and experience is needed to correctly identify and grade IGD, and to assess the ages at which IGD occurred.

In three cases (B005, B053, and B121), one episode of IGD is scored as less severe, and B061 as more severe by the experienced observer. Differences in IGD severity may have multiple causes. D’Ortenzio et al.^38^ describe the size of the interglobular spaces in Grade 3 as ‘large’ compared to the ‘moderate’ size of Grade 2, and a decrease in size, which could potentially be a result from the burning process, would therefore result in a lower Grade. In addition, the assessment of the amount of dentine that is affected relative to the surrounding normal dentine (expressed as < 25%, 25–50%, etc.) is another scoring aspect that determines the Grade of IGD. Snoddy et al.^43^ suggested the scoring method by D’Ortenzio et al.^38^ to be ambiguous, which would be exacerbated by the partial destruction of the burnt teeth, whereby only parts of the incremental dentine layers are preserved. This would also result in ‘under’- or ‘overscoring’ the percentage of the affected area. Although image analysis via grayscale histograms as proposed by Snoddy et al.^43^ decreases the risk of intra- and inter-individual errors, this method may be problematic to use due to the heat-induced deformation and damage to the dentine. Therefore, the visual scoring by D’Ortenzio et al.^38^ may be prone to some degree of interobserver errors (see Table 1), but remains a relatively easy and quick method for assessing IGD severity in burnt teeth that can be used by both experienced and inexperienced researchers. Therefore, it is unlikely that large differences in IGD severity can be attributed to the heating process and more likely are the result of slight variations in IGD appearance between the left and right molars of the same jaw and differences due to cutting the molars and canines at a slightly different location. Thus, the influence of temperature and duration on the appearance of IGD seems to be minimal and the assessment of IGD severity and the ages-of-occurrence in burnt dentine can successfully be undertaken in the vast majority of burnt teeth in this study.

**Figure 5:**
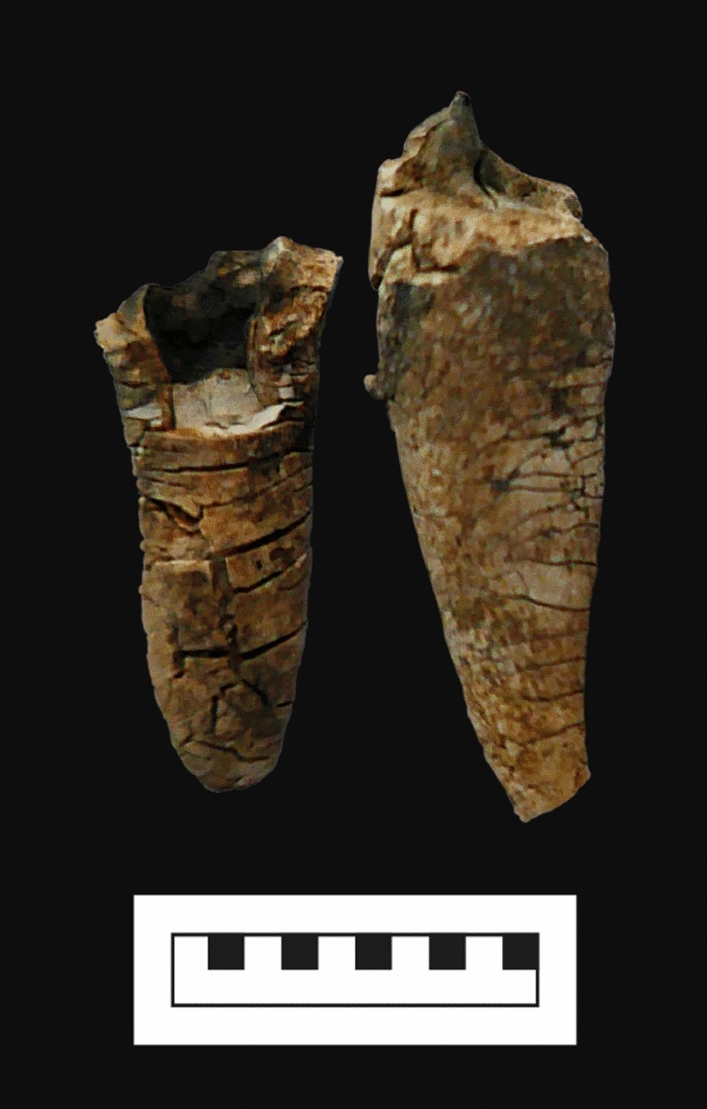
Burnt dental roots from an Early Medieval cremation deposit from Broechem, Belgium, showing a variation in burning degree.

Prior to this innovative study, it was suggested that temperatures of ≥ 900 °C obliterated all dental microstructures^40,41^. Consequently, no IGD would be visible in cremated human teeth, since the average temperature of outdoor pyres is postulated to be between 800 °C and 900 °C^39^, whereby experimental outdoor pyre cremations showed that temperatures of 1000 °C can also be reached for a short period of time^44^ and some may even reach higher temperatures^45^. However, this study clearly shows that IGD remains visible in dentine burned at 900 °C. In addition, even though a pyre may reach temperatures of ≥ 1000 °C, this is for a short period of time and the enamel and jawbone protects the dentine. In most cases, it can be advocated that the dentine is not exposed to these extreme temperatures (Fig. 5), which ensures IGD preservation and visibility.

The remarkable finding that IGD remains visible in cremated human teeth opens the way to document health conditions in vast number of past societies that regularly practiced cremation. Considering the results from just the burnt dentine, all the teeth that exhibited IGD had the first observable band around the age of 1 year. Commonly, the first vitamin D deficient episode occurs in the first year of a nonadult’s life, since the need for vitamin D during this period of significant growth is high^46^ and the vitamin D storage obtained from the mother during pregnancy will be depleted at approximately 8 weeks of age^47^. In more than half of the observable teeth with IGD (6/9; 66.7%), additional deficient episodes are observed at the age of about 2.5 years (see Fig. 3) or later (S095), indicating the disease was recurrent. Interestingly, all the teeth from the nineteenth–twentieth-century Koekelberg, Belgium (numbered with B) do not display additional bands of IGD after the age of about 2.5 years, suggesting their levels of vitamin D improved after that age of 2.5 years even though the need for vitamin D is postulated to be the same as in their first years of life^46^. However, the first molars will only record vitamin D deficient episodes up to the age of 10 years^48^. Additional bands of IGD may be found in the second and third molars, which cover later years in life^48^. Nevertheless, the postulated improvement in vitamin D levels after the age of 2.5 years in all the Koekelberg’s individuals suggest a change in child rearing practices and other sociocultural habits related to sunlight exposure, which was also observed in the collections from Beemster and Hattem, the Netherlands^3^, thus also improving our understanding of daily life of nonadults in industrialised Brussels, Belgium, in the nineteenth and twentieth centuries.

Demonstrating that some dental microstructures remain visible at high temperatures is tremendous step forward in the fields of biological anthropology and paleopathology. This innovative study enables the detection of vitamin D deficiency in cremated human remains, but also stimulates more bioarchaeological research to be undertaken on burnt human bone and teeth to further explore the visibility of other features that will increase our understanding of the influence environmental and sociocultural variables have on individuals. Furthermore, the differences in IGD appearance (i.e. partially fused calcospherites instead of dark semi-spherical spaces) that seemed to be linked to temperature and can further contribute to our understanding of variations in pyre temperatures, and pyre-management between and within past communities^44,49^ and may aid in the analysis of forensic contexts involving fire.

## Materials and methods

The molars used for this experimental research come from the ninetieth–twentieth century human skeletal collection Koekelberg from Brussels, Belgium and two canines come from the seventeenth–nineteenth century Broerekerk from Zwolle, the Netherlands were added to increase the number of teeth. The archaeological collection of Koekelberg was excavated in 2016 and consisted of more than 2000 comingled human remains. Before reburial in 2019, permission was obtained from Brussels Gewest, Belgium, to sample the available mandibulae and perform destructive analysis on the teeth. The archaeological collection of Broerekerk, excavated in the 90’s, was donated by the municipality of Zwolle to Leiden University, the Netherlands, upon which permission was obtained to do destructive analysis on one set of canines. Vitamin D deficiency is expected to be highly prevalent in the collection of Koekelberg, since Brussels was a large, highly industrialised urban centre in the nineteenth–twentieth centuries^50^. A total of 16 mandibles had at least the two first permanent molars (n = 32) available for sampling. One molar from each mandible (n = 16) and one mandibular canine were evaluated for IGD microscopically. For this purpose, the molars and the canine were cut using an IsoMet1000® precision saw producing thin sections of 70 µm and mounted on glass slides using the method as described by De Boer et al.^51^. The thin sections were investigated for IGD using a ZEISS® Axioscope 5 with a 50 × magnification. Assessment of IGD was undertaken in three steps. The 16 unburnt molars and one canine were evaluated for IGD, whereby the severity of IGD was estimated using the method of D’Ortenzio et al.^38^ distinguishing Grade 1 (small spaces and < 25% affected), Grade 2 (moderately large spaces and 25–50% affected), and Grade 3 (large spaces, > 75% affected). After this assessment, the mirroring molars and canine were divided into six groups and burned whole (not cut) in a muffle furnace at varying temperatures and durations (see Table 1, Results section). The 11 burnt teeth, which were suitable for further analysis, were embedded in Araldite® 2020 resin before sectioning after which a thin section was made as described above.

To assess not only the visibility of IGD after burning, but also the effect of high temperature on IGD severity, all groups contained molars and canines with varying degrees of IGD, from no IGD (as control) to Grade 3 (Table 1). Each of the burnt molars and canine was assessed for IGD visibility by observer BV (with extensive osteoarchaeological and IGD identification experience), thereby also evaluating severity, the number of deficient episodes, and the age(s) of occurrence using dental charts as developed by Brickley et al.^48^. The unburnt molars and canine were evaluated the same way to compare results between each pair of unburnt/burnt molars and canines (Table 1; 1st observation). This process was repeated again after a month to test repeatability (2nd observation). To evaluate the ease of IGD identification in burnt teeth, another observer (CS) with no osteoarchaeological or IGD identification experience was provided a short training by evaluating IGD in unburnt molars belonging to another collection. After this, the unburnt and burnt molars (n = 32) and the unburnt and burnt canines (n = 2) were investigated. Differences in age estimations of 0.5 year between observers BV and CS are considered to be minor.

Since the colour of most of retrieved dentine in archaeological cremation deposits is grey to white (see Fig. 5), the first set of teeth was burned at 600 °C for the duration of 2 h since this is the expected minimum temperature of an archaeological pyre, which could result in the observed colours^16,45^. The second set of teeth was burned at 800 °C (for 2 h), which is expected to be the average temperature of the pyres, based on the macroscopic assessment of the cremation material (i.e. colour, texture, etc.) and experimental pyres^39,44^. To test the influence of duration, the third set was burned at the same temperature (800 °C), but for 8 h. All bone and dentine microstructures are expected to have been obliterated at temperatures of ≥ 900 °C^40,52^. To test this, two molars were burnt at 900 °C for 2 h, two for 4 h, and one canine for 8 h.
